# Case report: “Major fetal cardiac pathology associated with a novel *CTNND1* mutation”

**DOI:** 10.3389/fped.2023.1180381

**Published:** 2023-05-19

**Authors:** Xuliang Zhao, Xu Li, Weiwei Sun, Zhuojun Wei, Min Yu, Man Zhang, Ruixia Tian

**Affiliations:** ^1^ Department of Laboratory, The 901th Hospital of the Joint Service of the People's Liberation Army, Hefei, China; ^2^ Department of Radiology, Anhui Children’s Hospital, Hefei, China; ^3^ Department of Medical, Beijing Chigne Translational Medicine Research Center, Beijing, China; ^4^ Department of Obstetrics and Gynecology, The 901th Hospital of the Joint Service of the People's Liberation Army, Hefei, China

**Keywords:** blepharocheilodontic syndrome, mutation, *CTNND1*, congenital heart disease, prenatal diagnosis

## Abstract

**Background:**

The p120-ctn protein, encoded by *CTNND1*, is involved in intercellular connections and regulates epithelial–mesenchymal transformation. *CTNND1* mutations can lead to blepharocheilodontic syndrome (BCDS). Increasing evidence shows that although BCDS mainly manifests as craniofacial and oral deformities, it can also present as congenital heart disease, limb deformities, and neurodevelopmental disorders.

**Case description:**

We report a prenatal case of a major cardiac malformation at 24^+3^ weeks of gestation. Ultrasound examination revealed a hypoplastic left ventricular, aortic coarctation, and a ventricular septal defect. Genetic analysis of the fetal tissues showed the presence of a novel mutation in *CTNND1* (NM_001085458.2: c.566_c.567insG; p.Pro190fs*15), which may lead to premature termination of protein coding, while both the parents harbored wild-type *CTNND1*. To date, only 15 *CTNND1* mutations have been reported in 19 patients worldwide, of which approximately 31% (6/19) had a cardiac phenotype.

**Conclusion:**

To the best of our knowledge, this is the first case report of fetal complicated cardiac malformations caused by this *CTNND1* mutation. Our findings provide new clinical references for prenatal diagnosis and suggest an important role for *CTNND1* in early cardiac development.

## 1. Introduction

Blepharocheilodontic syndrome (BCDS) is a rare autosomal dominant genetic disease, clinical manifestations of which consist of ectodermal dysplasia, such as eyelid deformity, cleft lip and palate, and abnormal teeth, as well as hypothyroidism, anal atresia, and neural tube defects caused by *CTNND1* mutations (1, 2). There are two subtypes of BCDS: BCDS1 (OMIM #119580), caused by *CDH1* mutations, and BCDS2 (OMIM #617681), caused by *CTNND1* mutations. These two genes may be involved in E-cadherin degradation and lead to BCDS, resulting in two BCDS subtypes with relatively similar phenotypes, both being mainly characterized by craniofacial deformities (3). Compared to the BCDS phenotype in humans, that in animal models can play a role in more extensive developmental abnormalities. For example, abnormal development of teeth, blood vessels, the kidneys, and the pancreas was observed in the *Ctnnd1* conditional knockout mouse model (4–6). Therefore, an increase in the number of reported patients with BCDS may enrich the knowledge of the disease phenotype. To date, less than 20 patients with *CTNND1* mutations have been reported. Alharatani et al. reported 13 patients with *CTNND1* mutations in nine families and expanded the range of phenotypes, with 6 patients presenting with congenital heart disease (CHD) of differing severities (7).

Here, we report a prenatal case of major fetal heart disease. The gene test results from fetal tissue after labor induction indicated a novel *CTNND1* frameshift mutation. The findings from this prenatal case provide clinical resources for prenatal diagnosis and genetic counseling and enrich the reported disease phenotypes and mutation spectrum.

## 2. Case description

**Figure 1:**
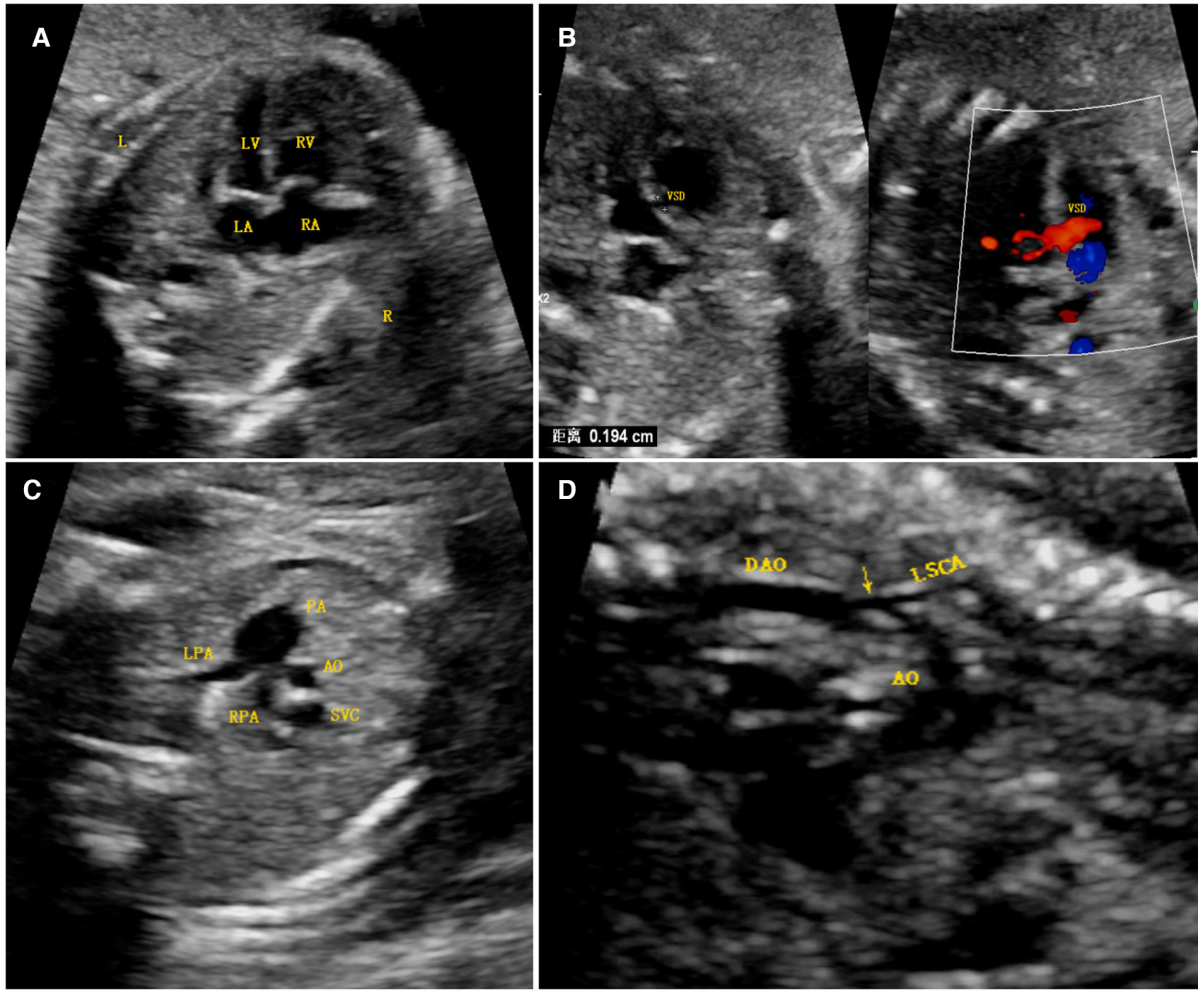
Antenatal ultrasound image. (**A**) The four-chamber view shows that the right ventricular cavity was significantly larger than the left cardiac cavity, the latter of which was still apex forming. (**B**) A ventricular septal defect was found just below the aortic outflow, as seen via two-dimensional imaging and color Doppler imaging. (**C**) A three-vessel view demonstrated a significantly larger main pulmonary artery relative to the aorta (PA > 1.6 AO), the latter of which was smaller than the superior vena cava. (**D**) A long-axis image of the aortic arch demonstrated diffuse hypoplasia of the arch to the isthmus and a posterior shelf (arrow), in keeping with the aortic coarctation. Abbreviations: LV, left ventricle; RV, right ventricle; LA, left atrium; VSD, ventricular septal defect; RA, right atrium; PA, pulmonary artery; LPA, left pulmonary artery; RPA, right pulmonary artery; SVC, superior vena cava; DAO, descending aorta; LSCA, left subclavian artery; AO, aorta.

A 25-year-old Han Chinese pregnant woman (first pregnancy) underwent an ultrasound examination at 24^+3^ weeks of gestation. The ultrasound results of the fetal heart showed a hypoplastic but apex-forming left ventricle, a hypoplastic aortic arch, coarctation of the aorta, and a ventricular septal defect (Figure 1). After induction of labor, fetal autopsy revealed ptosis and no obvious limb abnormalities, and the cardiac diagnosis was confirmed, per the suspicion arising after the ultrasound analysis.

Both parents were healthy and of Han Chinese ethnicity, were not close relatives, and denied a family history of congenital heart disease. They authorized the genetic testing of the fetal tissue to identify the pathogenic factors of this fetal CHD.

## 3. Diagnostic assessment

### 3.1. Subjects

We obtained written informed consent from the parents for publication of the clinical data, image map, and genetic mutation data of the fetus. All procedures were performed in accordance with the Declaration of Helsinki of 1964 and reviewed by the Ethics Committee of the 901st Hospital of the Joint Service of the People's Liberation Army (batch number 202112001).

### 3.2. Next-generation sequencing

We collected approximately 0.5 cm × 1.0 cm of skin tissue from the inner side of the fetus left leg; genomic DNA was extracted from the tissue samples, fragmented to an average size of 180–280 bp, and then used to create a DNA library according to the established Illumina paired-end scheme. The IDT xGen Exome Research Panel v2 (Integrated DNA Technologies, Coralville, IA, USA) was used for whole-exome sequencing, according to the manufacturer's instructions. The Illumina Novaseq 6,000 platform (Illumina Inc., San Diego, CA, USA) was used to sequence the genomic DNA with a minimum coverage depth of 10× and a target coverage of 99% (average coverage depth of 110×).

### 3.3. Genetic analysis

Offline data uses bcl2fastq v.2.20.0 software (Illumina Inc.) for basic call file conversion and demultiplexing. The BWA algorithm was used to align all data with the reference sequence (hg19/GRCh37). ANNOVAR (https://annovar.openbioinformatics.org/en/latest/user-guide/download/) was used to annotate the secondary allele frequencies, and toxicity and protection scores from the public dataset was used to filter and evaluate the possible pathogenicity of the variation. Variation was filtered according to public databases (dbSNP, 1,000 Genomes, and gnomAD), and that reported as pathogenic or possibly pathogenic in the Human Gene Mutation Database (http://www.hgmd.org/) and ClinVar database (https://www.ncbi.nlm.nih.gov/clinvar/) was retained. Online bioinformatics tools (SIFT, PolyPhen2, and MutationTaster) were used to predict the biohazards of the variation. Candidate pathogenic variants were identified according to the function, variation, and genetic pattern of the genes, and further evaluated according to the American College of Medical Genetics and Genomics guidelines (8).

### 3.4. Sanger sequencing validation

The primers for the exome-coding region of the target gene were designed using the Primer Premier software (version 5.0; Premier Bio-soft International, Palo Alto, CA, USA). The primer sequences for *CTNND1* were as follows: F, 5′-CCGGGAGAGACTGCCATAGTTA-3′; R, 5′-CTGTGACACGAGAGACAGTACA-3′. PCR was performed using PCR MasterMix polymerase (Tianjin Biotech, Beijing, China). PCR products were directly sequenced on an ABI 3,500 Genetic Analyzer (Applied Biosystems, Foster City, CA, USA).

### 3.5. A novel *CTNND1* mutation

**Figure 2:**
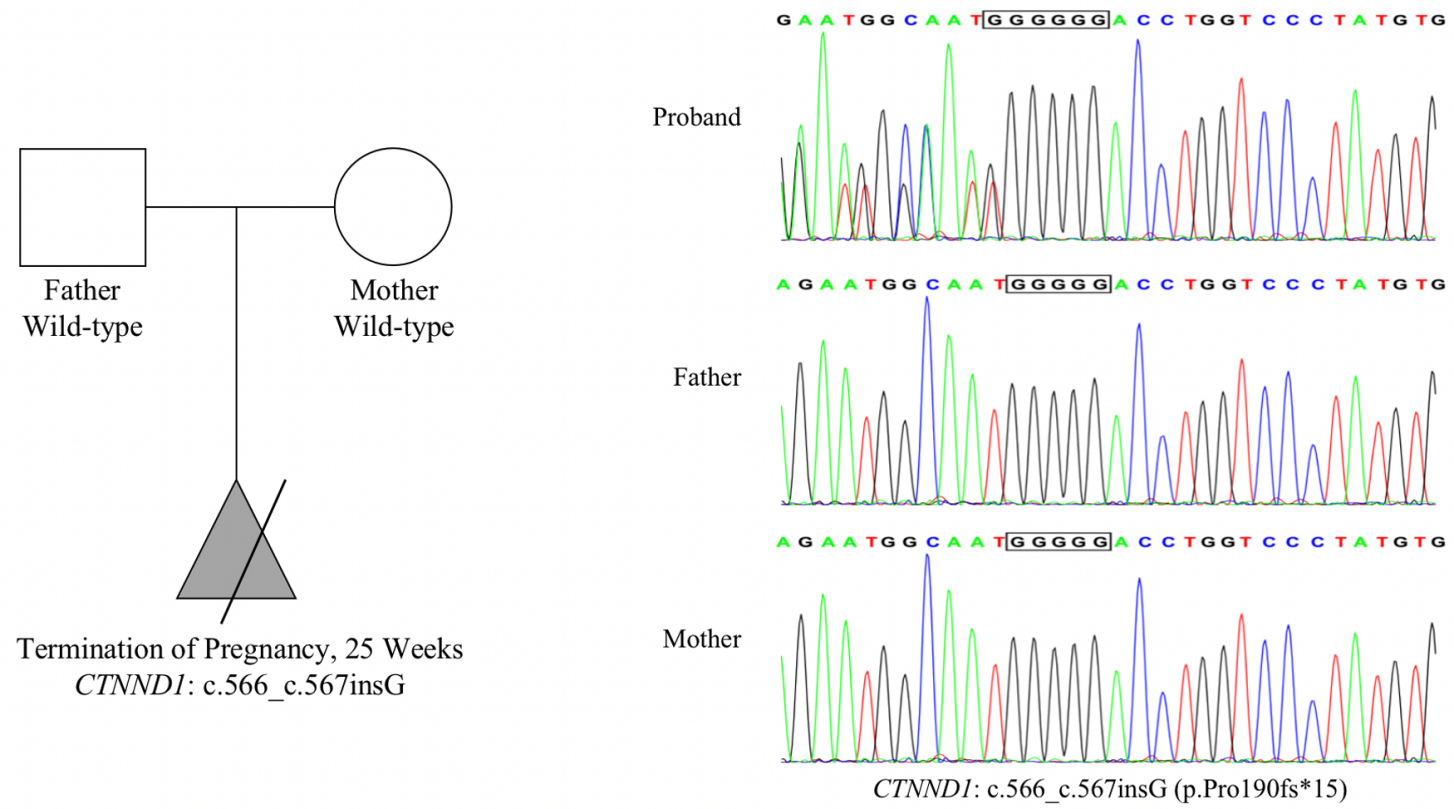
Mutation information. Whole-exome sequencing results show that the *CTNND1* in the proband had a frameshift mutation: NM_001085458.2: c.566_c.567insG (p.Pro190fs*15). Both parents harbored wild-type *CTNND1*, suggesting a *de novo* mutation. Sanger sequencing confirmed this mutation.

We identified a frameshift mutation in exon 6 of *CTNND1*, namely NM_001085458.2: c.566_c.567insG (p.Pro190fs*15), in the fetal tissue. Both parents carry wild-type *CTNND1*, which suggests a *de novo* mutation, but the possibility of low-abundance mutation in chimeric carriers cannot be ruled out. This mutation was not included in the dbSNP, 1,000 Genomes, or gnomAD databases, nor in the Human Gene Mutation Database or ClinVar database. According to the American College of Medical Genetics and Genomics guidelines, NM_001085458.2: c.566_c.567insG (p.Pro190fs*15) is a loss-of-function mutation, which may lead to loss of function (PVS1 level). This novel mutation (PS2 level) was confirmed using trio-based exome Sanger sequencing. The frequency of this mutation in the normal population is less than 0.0005 and has not been reported (PM2 level). Therefore, the NM_001085458.2: c.566_c.567insG (p.Pro190fs*15) mutation was classified as pathogenic, with the following rating evidence: PVS1 + PS2 + PM2. Sanger sequencing results confirmed the presence of this mutation in the fetus (Figure 2).

## 4. Discussion

Fetal CHD is one of the most common birth defects, with an incidence of approximately 4‰–5‰ in newborns (9). Single-gene mutations explain approximately 5% of CHD cases (9–11). In the present study, we report, to the best of our knowledge, the first instance of the prenatal diagnosis of major CHD, characterized by an apex-forming hypoplastic left ventricle, aortic coarctation, and a ventricular septal defect, in a fetus with a novel CTNND1 mutation (p.Pro190fs*15) that could be etiologic.

*CTNND1* is located on chromosome 11q12.1, consists of 21 exons, and generates 22 transcripts (https://www.ncbi.nlm.nih.gov/). Its encoded protein, p120-ctn, is an Armadillo-repeat protein that interacts with the intracellular domain of E-cadherin, stabilizes the trans-membrane domain of E-cadherin, and plays a role in adhesion and signal transduction between cells (12). In *Xenopus laevis*, *Ctnnd1* is highly expressed in the craniofacial bones and epidermis. In addition, p120-ctn plays an important role in the development of mouse eyelids and teeth (6, 13, 14). The main manifestations of human BCDS2 caused by *CTNND1* defects are oral abnormalities, including congenital tooth hypoplasia, retention of deciduous teeth, and delayed eruption of permanent teeth. Abnormal tooth morphology includes small/nail-shaped lateral incisors in permanent dentition and crown cracks in lateral incisors. Eyelid malformations are also characteristic phenotypes and include lower eyelid ectropion, upper eyelid deformity, and ptosis. In addition, some patients present developmental retardation, language retardation, and partial hypoplasia of the genitourinary system and corpus callosum (3, 7, 15, 16). Therefore, *CTNND1* is likely involved in different functions.

According to a previous study, BCDS mainly manifests as craniofacial malformations and oral abnormalities (3). However, subsequent studies have shown that *CTNND1* might be associated with CHD. In the BCDS cohort reported by Alharatani et al., the incidence of CHD was approximately 46% (6/13), including tetralogy of Fallot, aortic arch hypoplasia, aortic stenosis, ventricular septal defects, atrial septal defects, patent ductus arteriosus, and patent foramen ovale (7). Although the role of *CTNND1* in structural cardiac malformation remains unclear, it may be related to the development of the neural crest, which plays a crucial role in that of the cardiac diaphragm and valves (17). Indeed, Alharatani et al. showed that p120-ctn is highly expressed in human, mouse, and *Xenopus* embryonic hearts, and a functional defect in this protein might cause a cardiac phenotype by interfering with Wnt signal transduction in the neural crest (7).

To date, only 15 *CTNND1* mutations have been identified in 19 patients. The main types are frameshift and nonsense mutations, with only one splice mutation (Table 1). These mutations are all loss-of-function mutations and may produce a truncated p120-ctn protein. There seems to be no correlation between a *CTNND1* genotype and phenotype, which may be explained by the reduced number of reported patients with BCDS caused by *CTNND1* defects and the inability to screen them. However, Alharatani et al. observed that if the truncation mutation is located at the C-terminal of p120-ctn, complete cleft lip and cleft palate may appear, as opposed to the phenotype when its location is at the N-terminal regulatory region; therefore, they speculated that truncation mutations at the p120-ctn C-terminal cause a dominant negative effect (7). In agreement with the latter, the *CTNND1* p.Pro190fs*15 mutation reported in this study is located at the p120-ctn N-terminal regulatory region, and the fetus did not show a cleft lip or palate.

**Table 1:** Clinical phenotypes of patients harboring the *CTNND1* mutation.

No.	Variation (NM_00108558.1)	Amino acid variation	Mutation type	Eyelid deformity	Oral abnormality	Congenital heart disease	Neurodysplasia	Other	Ref.
1	c.606_627del	p.Pro203Leufs*25	Frameshift	+	+	–	–	Syndactyly	(3)
2	c.1093C > T	p.Gln365*	Nonsense	+	+	–	–	–	
3	c.2098C > T	p.Arg700*	Nonsense	+	+	–	–	–	
4	c.443_444delTG	p.Val148Aspfs*24	Frameshift	+	+	VSD, ASD, mitral valve stenosis, AAH	–	Scoliosis	(7)
5	c.443_444delTG	p.Val148Aspfs*24	Frameshift	+	+	VSD, ASD	Abnormal behavior	Dysplasia of the corpus callosum	
6	c.1381C > T	p.Arg461*	Nonsense	+	+	–	Autism spectrum disorder, intellectual disorder, hyperactivity disorder, behavioral abnormality	–	
7	c.1381C > T	p.Arg461*	Nonsense	+	+	VSD, ASD	Autism spectrum disorder	–	
8	c.2389C > T	p.Arg797*	Nonsense	+	+	–	–	–	
9	c.1481_1485del	p.Leu494Argfs*5	Frameshift	+	+	–	–	Limb abnormality, scoliosis, short stature, hyperthyroidism	
10	c.1481_1485del	p.Leu494Argfs*5	Frameshift	+	+	–	–	Limb abnormality	
11	c.1595del	p.Gly531Alafs*6	Frameshift	+	+	Tetralogy of Fallot	–	Limb abnormality, macroglossia	
12	c.2598_2601dupTGAT	p.Ser868*	Nonsense	+	+	–	–	–	
13	c.2598_2601dupTGAT	p.Ser868*	Nonsense	+	+	–	Autism spectrum disorder, intellectual disorder, hyperactivity disorder	–	
14	c.2702-5A > G	/	Splice	+	+	ASD, pulmonary stenosis, AAH	Autism spectrum disorder, intellectual disorder, hyperactivity disorder	Limb abnormality, short stature, cryptorchidism	
15	c.2737dupC	p.His913Profs*3	Frameshift	+	+	VSD	Intellectual disorder	Limb abnormality	
16	c.2737dupC	p.His913Profs*3	Frameshift	+	+	–	–	Limb abnormality, coronal hypospadias	
17	c.1372C > T	p.Arg458*	Nonsense	+	+	–	–	–	(16)
18	c.1595G > A	p.Gly532Asp	Frameshift	+	+	–	–	–	
19	c.2489G > A	p.Trp830*	Nonsense	+	+	–	–	–	
20	c.566_c.567insG	p.Pro190fs*15	Frameshift	+	NA	VSD, small left heart, ascending aorta, AAH	–	–	Our case

VSD, ventricular septal defect; ASD, atrial septal defect; AAH, aortic arch hypoplasia; +, occurrence; –, absence; NA, not applicable; *, termination codon.

In summary, we report a prenatal case of complex cardiac structural abnormalities at 24 ^+ 3^ weeks of gestation. Whole-exome sequencing and Sanger sequencing were performed using fetal tissues after labor induction. The results showed a novel *CTNND1* frameshift mutation, which may explain the early-onset CHD. We suggest *CTNND1* p.Pro190fs*15 as a pathogenic factor leading to fetal CHD, especially in the absence of typical craniofacial malformations or abnormal oral phenotypes. Our findings broaden the *CTNND1* mutation and phenotype spectra and reinforce the need for genetic testing at prenatal diagnosis in the face of complex cardiac phenotypes to improve diagnosis rates and genetic counseling during reproduction.

## Data Availability

The original data presented in the study are included in the article material; further inquiries can be directed to the corresponding author. The datasets presented in this study can be found in online repositories. The names of the repository/repositories and accession number(s) can be found below: ClinVar database repository, accession number SCV003845184 (https://www.ncbi.nlm.nih.gov/clinvar/).
